# Case report: Modified transplantation for pediatric patients with pyruvate kinase deficiency

**DOI:** 10.3389/fimmu.2024.1493398

**Published:** 2024-11-20

**Authors:** Yuhui Pang, Xiaoyu Qi, Jiayue Qin, Xiaoran Zhai, Rongxiao Wang, Jianzhu Cao, Na Zhang, Jinxia Liu, Jianying Li, Weihai Wu, Shanshan Wei, Jingjing Zhang, Shaofei Zhang, Yaochen Zhang, Yan Yue

**Affiliations:** ^1^ Department of Hematology, Shijiazhuang Pingan Hospital, Shijiazhuang, China; ^2^ Department of Medical Affairs, Acornmed Biotechnology Co., Ltd., Beijing, China; ^3^ Department of Pediatrics, Beijing United Family Women’s and Children’s Hospital, Beijing, China

**Keywords:** pyruvate kinase deficiency, allogeneic hematopoietic stem cell transplantation, modified conditioning regimen, severe transfusion dependence, case report

## Abstract

Pyruvate kinase deficiency (PKD) is an autosomal recessive genetic disease caused by mutations in the *PKLR* gene. To date, the clinical manifestations of PKD are heterogeneous, ranging from fetal anemia, neonatal jaundice, and severe chronic hemolytic anemia to fully compensated hemolytic anemia. Successful cases of allogeneic hematopoietic stem cell transplantation (allo-HSCT) for PKD have been reported, however, the number of cases is very small, and experiences are very limited. Here, we report two successful cases involving our modified conditioning regimen. This approach is suitable for patients with severe transfusion dependence. In conclusion, for PKD patients with severe transfusion dependence, allo-HSCT is an option and is currently a safe and effective way to completely eliminate the need for transfusions of drugs, such as Mitapivat, or genetic therapies and allow the patient to return to normal life.

## Introduction

Pyruvate kinase deficiency (PKD) is an autosomal recessive genetic disease caused by mutations in the *PKLR* gene. To date, the clinical manifestations of PKD are heterogeneous, ranging from fetal anemia, neonatal jaundice, and severe chronic hemolytic anemia to fully compensated hemolytic anemia (1, 2). Since its discovery in the 1960s, its clinical manifestations and pathogenesis have been fully elucidated, but there is no targeted treatment method at present (3, 4). In recent years, successful cases of allogeneic hematopoietic stem cell transplantation (allo-HSCT) for PKD have been reported, however, the number of cases is very small, and experience is very limited. Here, we report two successful cases involving our modified conditioning regimen.

## Case description

**Figure 1:**
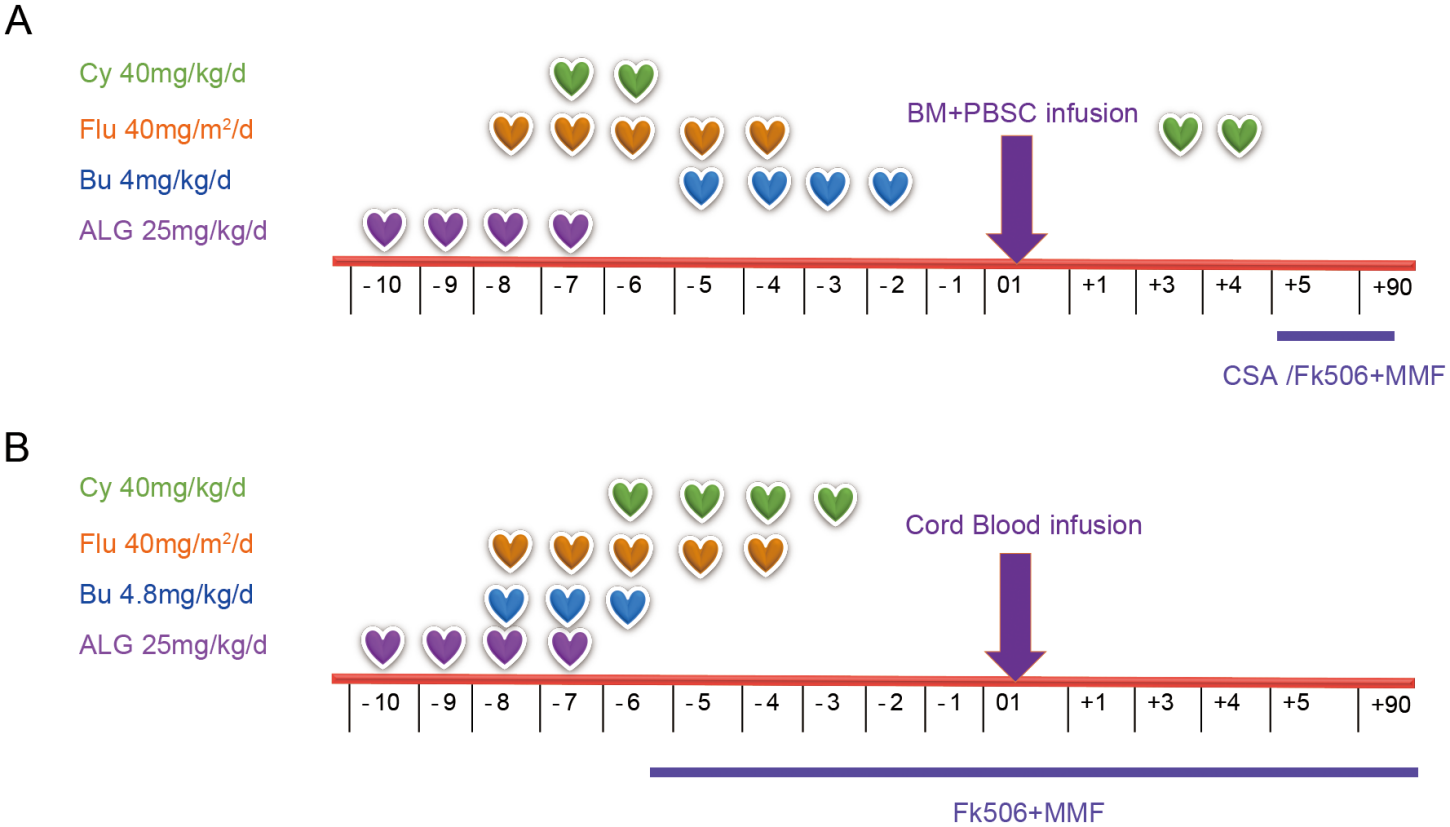
Conditioning regimens for patients with PKD, including Patient 1 **(A)** and Patient 2 **(B)**. PKD, pyruvate kinase deficiency; Cy, cyclophosphamide; Flu, fludarabine; Bu, Busulfan; ALG, antilymphocyte globulin; BM, bone marrow; PBSC, peripheral blood stem cell; CSA, cyclosporine A; MMF, mycophenolate mofetil.

Case 1 is a male patient, aged 3 years and 6 months, who was admitted to the hospital on July 19, 2019. The patient was found to be pallid, jaundiced (yellow stain on the sclera), anemic and to have an enlarged spleen. He was markedly anemic, with a Hb level of 5 g/dl. His RBC glucose-6-phosphate dehydrogenase (G-6-PD) level (PK activity) was 12% and Hb typing revealed E F A (20.2, 15.1, 66.7%). His serum ferritin level was 2131.62 ng/ml prior to HSCT. Iron chelation was used and HSCT was performed after the serum ferritin level reached a normal level. Several packed red cell transfusions were given to improve the symptoms of anemia, and a compound heterozygous mutation in the *PKLR* gene was detected via whole exome sequencing, with one copy from each of the patient’s parents (his father: chr1:155261637, c.1528C>T, p.R510X; his mother: chr1:155264297, c.941T>C, p.I314T) as summarized in **Table 1**. Before transplantation, support treatments were given, and blood transfusions were performed every 2–3 weeks before HSCT. Before transplantation, the patient was given 700 ml of replacement plasma to ameliorate his jaundice. There were no suitably matched donors. His father human leukocyte antigen (HLA 6/12), aged 32 years, was a haplo-identical donor. HSCT was performed in August 24, 2021. The boy received busulfan (Bu) (4.8 mg/kg/day × 4 days) from Days -5 to -2, cyclophosphamide (Cy) (40 mg/kg/day × 2 days) from Days -7 to -6, fludarabine (Flu) (40 mg/m2/day × 5 days) from Days -6 to -2, and antilymphocyte globulin (ALG, Wuhan Biological Products Research Institute Co., Ltd., 25 mg/kg/d × 4 days) from Days -10 to -7. Tacrolimus (0.01 to 0.05 mg/kg/day) was initially administered on Day +5 with the intention of tapering off by Day +90 if there was no graft-versus-host disease (GvHD). Mycophenolate mofetil (MMF) was administered on Day +5 and tapered off by Day +28. The patient received methotrexate (10 mg/m^2^/day) on Days +1, +3, +6, and +11. The modified posttransplant cyclophosphamide (PT/Cy) conditioning regimen is detailed in **Figure 1A** and **Table 1**. The grafts were both bone marrow (BM) and peripheral blood stem cells (PBSCs) mobilized with recombinant human granulocyte colony stimulating factor (G-CSF). The PBSC doses used were 18×10^8^ TNC/kg, 18×10^6^ CD34/kg, and 4.3×10^8^ CD3/kg, and the BM doses used were 3.2×10^8^ TNC/kg, 0.96×10^6^ CD34/kg, and 0.15×10^8^ CD3/kg.

**Table 1:** Patient characteristics.

	Case 1	Case 2
Sex	Male	Female
*PKLR* genotype	His father: chr1:155261637, c.1528C>T, p.R510X; his mother: chr1:155264297, c.941T>C, p. I314T	Her father: chr1:155261637, c.1528C>T, p.R510X; her mother: chr1:155264940, c.661G>A, p. D221N
Splenectomy	Intermediate	Intermediate
Age at HSCT	3.5 years	3.3 years
Pre-transplant ferritin	2131.62 ng/ml	491.02 ng/ml
Donor	Father	Cord blood
Conditioning Regimen	Flu/Bu/Cy/ALG	Flu/Bu/Cy/ALG
Matching	HLA 6/12	HLA 7/10
Stem cell source	BM + PBSC	Cord blood
GvHD	No	aGvHD (I° Skin)
Infection	No sever infection	No sever infection
Outcome	Well and alive	Well and alive
Follow-up time (Months)	62	34

HSCT, hematopoietic allogeneic stem cell transplantation; GvHD, graft-versus-host disease; aGvHD, acute graft-versus-host disease; Flu, fludarabine; Bu, busulfan; Cy, cyclophosphamide; ALG, antilymphocyte globulin; BM, bone marrow; PBSC, peripheral blood stem cell.

The granulocytes were successfully engrafted on Day 14 after transplantation, and the platelets were engrafted on Day 11. The final red blood cell transfusion was performed on Day 12 after the transplantation. The chimerism rate after transplantation was 100%, without complications such as GvHD or severe infection. On 27 months after transplantation, all immunosuppressors had been discontinued, and the patient’s hemoglobin level was found to be normal during the routine follow-up. On re-evaluation of the RBC PK of the patient at 1.5 years posttransplant, the PK activity was 118% that of the normal control, whereas the pretransplant level was 12% in this patient. After HSCT, the serum ferritin level was 343.6 ng/ml, which was normal. The patient is currently healthy and normal physically and socially.

Case 2 is a female patient, who was diagnosed with PKD by genetic testing, and like Patient 1, she was diagnosed at the age of 4 years and 3 months on December 22, 2021, due to jaundice and anemia at birth. The results revealed that the *PKLR* gene complex contained a compound heterozygous mutation (her father: chr1:155261637, c.1528C>T, p.R510X; her mother: chr1:155264940, c.661G>A, p.D221N) as shown in **Table 1**. Her PK activity was normal before transplantation. Her serum ferritin level was 491.02 ng/ml prior to HSCT. During the 4-year course of her illness, blood transfusion support was used when the patient’s hemoglobin level decreased to approximately 50 g/L, which occurred approximately once a month before transplantation. Upon admission, the physical examination revealed an anemic appearance, yellow sclera and splenomegaly. The patient received a plasma exchange to improve her liver function, as the T-bil level was 146 µmol/L. As in Case 1, there was no matched sibling donor, and nonconsanguineous cord blood (HLA 7/10, blood type AB+) stem cells were transfused. The cord blood cell dose was 6.5×10^7^ TNC/kg, 3.74×10^6^ CD34/kg as detailed in **Table 1**.

The improved conditioning regimen was as follows: Bu (4.8 mg/kg/day × 3 days) from Days -8 to -6, Cy (40 mg/kg/day × 4 days) from Days -6 to -3, Flu (40 mg/m2/day × 5 days) from Days -8 to -4, and ALG (Wuhan Biological Products Research Institute Co., Ltd., 25 mg/kg/d × 4 days) from Days -10 to -7, as shown in detail in **Figure 1B** and **Table 1**. This patient developed cyclosporin-related encephalopathy and received tacrolimus prophylaxis for GvHD. Twenty-three days after transplantation, the granulocytes were engrafted. The platelets were engrafted on Day 51. The last red blood cell transfusion was performed on Day 44 after transplantation, and ABO blood type conversion was complete on Day 53. After HSCT, her serum ferritin level was 3520.53 ng/ml due to RBC transfusion during transplantation, iron chelation was used, and her serum ferritin level was normal at 6 months after transplantation. The posttransplant chimerism rate was 100%, and only acute I° skin GvHD occurred, which was cured after the topical application of dexamethasone cream. From follow-up until 22 months after transplantation, the patient’s hemoglobin level remained normal, and she was able to return to school.

## Discussion

Here, we have described the successful management of two patients with transfusion-dependent PKD with allo-HSCT via a modified conditioning regimen. Iron overload may also be due to ineffective erythropoiesis and low hepcidin levels, increased intestinal absorption, or coinheritance of hemochromatosis genes. Iron overload is observed at all ages and in regularly transfused, non-transfused and infrequently transfused patients. The risk of iron loading is lifelong and influences the result of HSCT (5, 6). In these 2 cases, prior to HSCT, Patient 1 underwent iron chelation via the administration of oral deferasirox at an iron level of less than 1000 µg/L, and HSCT was initiated.

Animal models have been used in experiments to confirm that HSCT may virtually cure PKD (7), and since guidelines for the use of HSCT in PKD patients are lacking, our findings may be a helpful first step toward the development of protocols in the future. Compared with the published survival rates for other forms of hereditary anemias in cohorts that are otherwise comparable in age, time period and transplant hospital, the overall survival rate after HSCT in PKD patients is relatively low. In a larger series of 16 patients undergoing HSCT in Europe and Asia, 74% cumulative survival was reported, although there was a nontrivial rate of GvHD (8). In our study, Patient 1 did not have a suitable donor, and a modified conditioning regimen combined with PT/Cy as GvHD prophylaxis achieved good results. Severe acute GvHD and chronic GvHD were not observed in this patient; furthermore, ALG was used for the first time in PKD patients, and the dose and potency were based on our center’s experiences (9). In Patient 2, cord blood was used as an alternative source, and the advantage of cord blood is obvious; the less stringent HLA matching required makes it easier to find a donor across HLA barriers, severe infection and GvHD didn’t occurred in this patient after HSCT (10).

In the above 2 cases, allo-HSCT was shown to cure PKD, and its effectiveness is clear. This approach is suitable for patients with severe transfusion dependence. As Case 1 was absence of a suitable donor, we used his father who carried gene mutation but normal hemoglobin as a more suitable donor. After HSCT, the patient ‘s hemoglobin level returned to normal and reached complete chimerism. In both 2 cases, with the use of this modified transplant protocol, we didn’t observe the occurrence of hepatic veno-occlusive diseas and transplantation associated thrombotic microangiopathy. However, this program has not been reported by other medical institutions, and its long-term efficacy needs to be supported by continuous follow-up and more case data. To control the risk of transplantation-related complications, it is recommended that such patients be treated in hospitals with mature transplantation technology, especially with respect to conditioning regimens, the number of stem cell transfusions, donor selection and other aspects. With regard to severe GvHD, prevention is the main approach.

In conclusion, for PKD patients with severe transfusion dependence, allo-HSCT is an option and is currently an effective and potential way to completely eliminate the need for patients with PKD transfusion dependent and return to normal life.

## Data Availability

The raw data supporting the conclusions of this article will be made available by the authors, without undue reservation.
